# Infection with *Anaplasma phagocytophilum *in a seronegative patient in Sicily, Italy: Case Report

**DOI:** 10.1186/1476-0711-4-15

**Published:** 2005-10-03

**Authors:** J de la Fuente, A Torina, V Naranjo, S Caracappa, V Di Marco, A Alongi, M Russo, AR Maggio, KM Kocan

**Affiliations:** 1 Department of Veterinary Pathobiology, Center for Veterinary Health Sciences, Oklahoma State University, Stillwater, OK 74078, USA; 2 Instituto de Investigación en Recursos Cinegéticos IREC (CSIC-UCLM-JCCM), Ronda de Toledo s/n, 13005 Ciudad Real, Spain; 3 Intituto Zooprofilattico Sperimentale della Sicilia, Via G. Marinuzzi n° 3, 90129 Palermo, Italy; 4 Istituto Zooprofilattico Sperimentale della Sicilia, Area Barcellona Pozzo di Gotto, Via S. Andrea n° 96, 98051 Barcellona, Italy

**Keywords:** Human granulocytic anaplasmosis, *Anaplasma phagocytophilum*, 16S rDNA sequence

## Abstract

**Background:**

*Anaplasma phagocytophilum *causes human granulocytic anaplasmosis (HGA) in humans, which has been recognized as an emerging tick-borne disease in the United States and Europe. Although about 65 cases of HGA have been reported in Europe, some of them do not fulfill the criteria for confirmed HGA. Confirmation of HGA requires *A. phagocytophilum *isolation from blood, and/or identification of morulae in granulocytes and/or positive PCR results with subsequent sequencing of the amplicons to demonstrate specific rickettsial DNA. Seroconversion or at least fourfold increase in antibody titers to *A. phagocytophilum *has been used as criteria for confirmed HGA also.

**Case presentation:**

Infection with *A. phagocytophilum *was confirmed by PCR in a patient in Sicily, Italy, who had negative serology for *A. phagocytophilum*. A fragment of *A. phagocytophilum *16S rDNA was amplified by two independent laboratories and sequenced from two separate patient's blood samples. The 16S rDNA sequence was identical in both samples and identical to the sequence of the *A. phagocytophilum *strain USG3 originally obtained from a dog.

**Conclusion:**

Infection with *A. phagocytophilum *was confirmed in a patient without a detectable antibody response against the pathogen. The results reported herein documented the first case of confirmed HGA in Sicily, Italy. These results suggested the possibility of human infections with *A. phagocytophilum *strains that result in clinical symptoms and laboratory findings confirmatory of HGA but without detectable antibodies against the pathogen.

## Background

*Anaplasma phagocytophilum *(Rickettsiales: Anaplasmataceae) causes human granulocytic anaplasmosis (HGA) in humans, which has been recognized as an emerging tick-borne disease in the United States and Europe [1,2]. The disease was first described in the United States in 1994 [3,4] and in Slovenia in 1997 [5]. About 65 cases have been reported in Europe although some of them do not fulfill the criteria for confirmed or probable HGA [2]. Human infection with *A. phagocytophilum *could be confirmed by identification of morulae in granulocytes, positive PCR results using whole blood as a substrate, and/or isolation of *A. phagocytophilum *from the blood [2]. Serological tests, particularly indirect immunofluorescence assay (IFA), are commonly used but are often negative during the initial phase of the disease. However, high and stable antibody titers to *A. phagocytophilum *are usually found in patients with probable HGA who show clinical signs and symptoms that most likely are not the result of a recent infection with *A. phagocytophilum *[2].

Although the seroprevalence of HGA in central and southern Italy is high in workers at risk for tick bites [6], only two cases of HGA have been reported in Italy [7]. However, the analysis of *A. phagocytophilum *DNA was not performed in these cases. The objective of this study was to characterize the 16S rDNA sequence of a strain of *A. phagocytophilum *obtained from a seronegative patient with confirmed HGA in Sicily, Italy.

## Case Presentation

### Patient, materials and methods

On May 8, 2004, a 51-year-old man living in Barcellona Pozzo di Gotto, Messina, Sicily, Italy was admitted to the hospital with a 3-month fever (37.2°C), arthralgia, myalgia, malaise, pallor, stiffnes of hands and knee, nausea, low appetite and weight loss. The patient did not recall a tick bite but he visited hunting areas 15 days before the fever started and kept exotic birds (golden pheasants, partridges and guinea fowls) at his house for two years. The patient showed abnormal liver (462 mU/ml alkaline phosphatase, normal range (nr) = 98–280; 1.20 mg/dl total bilirubin, nr = 0.16–1.10; 0.35 mg/dl direct bilirubin, nr = 0.0–0.2) and renal (1.5 mg/dl creatinine, nr = 0.5–1.2) functions and low hemoglobin (9.9 g/dl; nr = 12–18). Pulmonary, cardiac and immunological involvement was absent. Cell counts and serum C-reactive protein levels were normal.

On September 13, 2004 the patient was admitted to the hospital for a second time. In addition to previous findings, the patient showed a mild splenomegaly, hemorrhoids, osteopenia, hiatal hernia, gastritis and inflammation of duodenum. Systemic inflammatory pathology and neoplasia were ruled out. Doctors suspected a depressive syndrome and the patient was treated with antidepressants.

Serum antibodies were determined for Lyme borreliosis, *Rickettsia conorii*, *Coxiella burnetii *and *A. phagocytophilum *(Fuller Laboratories, Fullerton, CA, USA), and *Ehrlichia chaffeensis *and *A. phagocytophilum *(Focus Technologies, Cypress, CA, USA) following manufacturer's recommendations. FITC-conjugated anti-human IgG (Sigma, St. Louis, MO, USA) and IgM (Aliphadia S.A., Wavre, Belgium) were used as secondary antibodies. The immunofluorescence tests for *A. phagocytophilum *use antigens derived from human HL-60 cells infected with the HGE-1 isolate and samples were considered negative when no fluoresce was detected at 1:80 (Fuller Laboratories) or 1:64 (Focus Technologies) dilution of the test serum, as recommended by the manufacturer. DNA was purified using GenElute Mammalian Genomic DNA Miniprep Kit (Sigma) or TriReagent (Sigma) from patient's blood samples collected in September 8 and October 8, 2004. PCR analysis for *R. conorii*, *C. burnetii*, *E. chaffeensis*, *Theileria *spp., *Babesia *spp. and *Anaplasma *spp. were conducted on both DNA samples as previously described [8-11]. PCRs were conducted with 1 μl (0.1–10 ng) DNA using 10 pmol of each primer in a 50-μl volume (1.5 mM MgSO_4_, 0.2 mM dNTP, 1 × AMV/Tfl 5 × reaction buffer, 5u *Tfl *DNA polymerase) employing the Access RT-PCR system (Promega, Madison, WI, USA). Reactions were performed in an automated DNA thermal cycler for 35 cycles. PCR products were electrophoresed on 1% agarose gels to check the size of amplified fragments by comparison to a DNA molecular weight marker (1 Kb DNA Ladder, Promega). Control reactions were done without the addition of DNA to rule out contaminations during PCR.

Amplified *Anaplasma *16S rDNA fragments were resin purified (Wizard, Promega) and cloned into pGEM-T vector (Promega) for sequencing both strands by double-stranded dye-termination cycle sequencing (Core Sequencing Facility, Department of Biochemistry and Molecular Biology, Noble Research Center, Oklahoma State University). Two independent clones were sequenced from each PCR. Multiple sequence alignment was performed with the program AlignX (Vector NTI Suite, version 5.5; InforMax, North Bethesda, MD, USA).

## Results and discussion

The analysis of the clinical symptoms and laboratory results of the patient described herein suggested the possibility of an infectious agent as the cause of the illness. Viral infections were ruled out and the investigation was directed towards the identification of tick-borne pathogens.

Serology for Lyme borreliosis, *R. conorii*, *C. burnetii*, *E. chaffeensis *and HGA and PCR analyses for *R. conorii*, *C. burnetii*, *E. chaffeensis*, *Theileria *spp. and *Babesia *spp. were negative. However, positive PCR results were obtained for *Anaplasma *spp. in patient's blood samples collected in September 8 and October 8, 2004. PCR reactions for *Anaplasma *spp. 16S rDNA were conducted with independent DNA extractions in two laboratories (in Palermo, Sicily and in Ciudad Real, Spain) to confirm the results. Control reactions ruled out PCR contaminations and the sequence of the 16S rDNA fragment obtained from the patient (Genbank accession number DQ029028) was identical in both DNA samples and identical to the sequence of the *A. phagocytophilum *strain USG3 originally obtained from a dog (Genbank accession number AY055469; ref. [12]).

The patient described herein fulfilled the criteria for confirmed HGA, including prolonged fever, arthralgia, myalgia, malaise, nausea, abnormal liver function and positive PCR and sequence analysis for *A. phagocytophilum *DNA. However, the patient was seronegative for up to six months after detection of pathogen infection by PCR. These results suggested that the patient presented a chronic stage of infection with symptoms produced by a secondary illness related or not to *A. phagocytophilum *and/or could has been infected with a strain of the pathogen that could not be detected by existing serological tests or induces a low antibody response. Strain genetic differences have been reported in *A. phagocytophilum *and may be associated with variations in pathogenicity and host tropism [10,12-14], although the exact relationship between these factors is presently unknown.

## Conclusion

The results reported herein demonstrated a prolonged *A. phagocytophilum *infection in a patient without a detectable antibody response against the pathogen. These results documented the first case of prolonged *A. phagocytophilum *infection in Sicily, Italy and suggest the possibility of human infections with *A. phagocytophilum *strains that result in clinical symptoms and laboratory findings confirmatory of HGA but without detectable antibodies against the pathogen.

## Authors' contributions

JF carried out the sequence analysis, designed the study and drafted the manuscript. AT and SC conceived the study, and participated in its design and coordination and helped to draft the manuscript. VN and AA carried out the molecular genetic studies. VDM and MR collected and analyzed the clinical data. ARM carried out the immunoassays. KMK helped to draft the manuscript. All authors read and approved the final manuscript.
